# Clinical application of digital technology in the use of anterolateral thigh lobulated perforator flaps to repair complex soft tissue defects of the limbs

**DOI:** 10.1093/burnst/tkae011

**Published:** 2024-05-10

**Authors:** Kai-xuan Dong, Ya Zhou, Yao-yu Cheng, Hao-tian Luo, Jia-zhang Duan, Xi Yang, Yong-qing Xu, Sheng Lu, Xiao-qing He

**Affiliations:** Department of Orthopedics, The First People’s Hospital of Yunnan Province, The Affiliated Hospital of Kunming University of Science and Technology, Key Laboratory of Digital Orthopedics of Yunnan Province, 157 Jinbi Road, Xishan District, Kunming, Yunnan 650032, China; School of Rehabilitation, Kunming Medical University, 1168 Chunrong west Road, Yuhua Street, Chenggong District, Kunming, Yunnan 650504, China; Department of Orthopedics, The First People’s Hospital of Yunnan Province, The Affiliated Hospital of Kunming University of Science and Technology, Key Laboratory of Digital Orthopedics of Yunnan Province, 157 Jinbi Road, Xishan District, Kunming, Yunnan 650032, China; Department of Orthopedics, The First People’s Hospital of Yunnan Province, The Affiliated Hospital of Kunming University of Science and Technology, Key Laboratory of Digital Orthopedics of Yunnan Province, 157 Jinbi Road, Xishan District, Kunming, Yunnan 650032, China; Affiliated Hospital of Yunnan University, 176 Qinnian Road, Wuhua District, Kunming, Yunnan 650032, China; Department of Orthopedics, 920^th^ Hospital of the Joint Logistic Support Force, People's Liberation Army of China, 212 Road, Daguan District, Kunming, Yunnan 650032, China; Department of Orthopedics, 920^th^ Hospital of the Joint Logistic Support Force, People's Liberation Army of China, 212 Road, Daguan District, Kunming, Yunnan 650032, China; Department of Orthopedics, The First People’s Hospital of Yunnan Province, The Affiliated Hospital of Kunming University of Science and Technology, Key Laboratory of Digital Orthopedics of Yunnan Province, 157 Jinbi Road, Xishan District, Kunming, Yunnan 650032, China; Department of Orthopedics, 920^th^ Hospital of the Joint Logistic Support Force, People's Liberation Army of China, 212 Road, Daguan District, Kunming, Yunnan 650032, China

**Keywords:** Digital technology, Anterolateral thigh lobulated perforator flap, Complex limb defect, Reconstruction, Microsurgery

## Abstract

**Background:**

It is challenging to repair wide or irregular defects with traditional skin flaps, and anterolateral thigh (ALT) lobulated perforator flaps are an ideal choice for such defects. However, there are many variations in perforators, so good preoperative planning is very important. This study attempted to explore the feasibility and clinical effect of digital technology in the use of ALT lobulated perforator flaps for repairing complex soft tissue defects in limbs.

**Methods:**

Computed tomography angiography (CTA) was performed on 28 patients with complex soft tissue defects of the limbs, and the CTA data were imported into Mimics 20.0 software in DICOM format. According to the perforation condition of the lateral circumflex femoral artery and the size of the limb defect, one thigh that had two or more perforators from the same source vessel was selected for 3D reconstruction of the ALT lobulated perforator flap model. Mimics 20.0 software was used to visualize the vascular anatomy, virtual design and harvest of the flap before surgery. The intraoperative design and excision of the ALT lobulated perforator flap were guided by the preoperative digital design, and the actual anatomical observations and measurements were recorded.

**Results:**

Digital reconstruction was successfully performed in all patients before surgery; this reconstruction dynamically displayed the anatomical structure of the flap vasculature and accurately guided the design and harvest of the flap during surgery. The parameters of the harvested flaps were consistent with the preoperative parameters. Postoperative complications occurred in 7 patients, but all flaps survived uneventfully. All of the donor sites were closed directly. All patients were followed up for 13–27 months (mean, 19.75 months). The color and texture of each flap were satisfactory and each donor site exhibited a linear scar.

**Conclusions:**

Digital technology can effectively and precisely assist in the design and harvest of ALT lobulated perforator flaps, provide an effective approach for individualized evaluation and flap design and reduce the risk and difficulty of surgery.

Highlights

- The ALT lobulated perforator flap is the ideal choice for repairing wide or irregular defects of the limbs. However, the flap design and harvesting processes are demanding and the failure rate is high.
- A certain length between the selected perforators to allow the two lobes of the flap to move relatively freely is very important for the ALT lobulated perforator flap.
- Computed tomography angiography allows accurate visualization of the vascular anatomy of the flap, which is key to successful skin flap transplantation.
- Digital technology can effectively facilitate accurate preoperative design and individualization and guide the intraoperative harvesting of ALT lobulated perforator flaps. This technology can improve the accuracy and success rate of the operation and offers unique advantages for clinical application.

## Background

Soft tissue defects in limbs can be caused by trauma and are often accompanied by the exposure of tendons, bones and other deep tissues. The main treatment method for these defects is skin flap repair. The increasing number of patients with complex defects, especially wide or irregular defects, is a challenge in the field of reconstructive surgery [1–3]. Several methods have been reported for the reconstruction of complex limb defects [4–8], including pedicled fasciocutaneous flaps, microsurgical free flaps and multiple flaps, among others. However, because of the limited amount of soft tissue available and the less-versatile design of conventional flaps, the transfer of multiple flaps requires a flow-through flap to revascularize the second free flap, which is more time consuming due to the need for additional microsurgical anastomosis and leads to higher donor site morbidity due to the need to harvest the additional flap [9, 10]. Thus, this approach is not the best choice for the reconstruction of such complex defects.

Since Song *et al*. [11] introduced the anterolateral thigh (ALT) flap, it has become one of the most popular choices for reconstructive surgeons in the repair of complex defects caused by trauma because it can provide a large area of skin, cause minimal damage to the donor site, provide a long vascular pedicle and avoid sacrificing major blood vessels [12–15]. The ALT lobulated perforator flap is a special type of ALT flap in which each lobe is supplied by a separate perforator, both of which originate from the same source vessel (donor area); with these flaps, only one set of vascular pedicles needs to be anastomosed during the transplantation process to rebuild the blood circulation in two or more flap lobes [16] for the repair of wide or irregular defects. However, there is great variation in the source and number of perforators [17], and stringent design requirements exist for the ALT lobulated perforator flap [18]. Even for experienced surgeons, harvesting a lobulated perforator flap is still a major challenge. With the development of digital imaging technology and computer image processing technology, digital technology is increasingly used for broad applications in the medical field. The application of this technology to assist in the use of bone flaps for repair and reconstruction has been reported previously [19, 20]. Digital technology for reconstruction of the flap vasculature can be used to optimize the surgical strategy preoperatively, which may be very useful for the design and harvest of lobulated perforator flaps.

To achieve accurate preoperative acquisition of flap vascular anatomical information, perforator localization, precise flap design and reduced surgical risk, we incorporated digital technology into the application of ALT lobulated perforator flaps for the reconstruction of complex defects of the extremities. From December 2013 to February 2022, we used ALT lobulated perforator flaps to repair wide or irregular complex extremity defects in 28 patients. Before surgery, digital technology was used for 3D visual reconstruction of the flap. The flap vascular anatomical information and design were used to guide the incisions for harvesting the ALT lobulated perforator flap during the operation, with good curative efficacy. The purpose of this study was to review the use of digital technology in the use of ALT lobulated perforator flaps for repairing complex defects of the extremities and to report our experience in the clinical repair of such complex defects.

### Data and patients

From December 2013 to February 2022, 28 patients with complex defects of the extremities underwent reconstruction surgery with ALT lobulated perforator flaps using a digital technology-assisted technique; these patients included 19 males and 9 females aged 15–61 years (mean, 39.39 years). The defects were caused by trauma in 25 patients and by diabetes in 3 patients. All patients in this series had wide or irregular soft tissue defects with tendon, bone or nerve exposure. The defect size ranged from 12 × 10 cm to 18 × 17 cm. The area of involvement was the leg in 2 patients, the ankle/foot in 15 patients, the upper arm in 1 patient, the elbow in 1 patient and the hand in 9 patients (Table 1). Debridement and vacuum sealing drainage (VSD) were performed in the first stage after admission, and patients with fractures were simultaneously fixed with a Kirschner wire or external fixator. All patients signed informed consent forms.

**Table 1:** Patient demographics (n = 28)

	**Number**
Male	19
Female	9
Cause	
Machine injury	16
Motor vehicle accident	9
Diabetes	3
Location	
Foot and ankle	15
Leg	2
Hand	9
Elbow	1
Upper arm	1
Skin defect size (cm × cm)	12 × 10–18 × 17

### Image acquisition

All patients were examined via computed tomography angiography (CTA) before the operation. A Siemens SOMATOM Force (Germany) third-generation dual-source CT scanner was used for continuous scanning of the area from the lower segment of the abdominal aorta to the bilateral anterior tibial artery, posterior tibial artery and peroneal artery. The CTA acquisition parameters are shown in Table 2.

**Table 2:** Computed tomography angiography (CTA) acquisition parameters

**Parameter**	
Gantry rotation time (s/r)	0.5
Tube voltage (kV)	70
Tube current modulation	Automated tube current (CARE Dose 4D)
Matrix	512 × 512
Pitch	0.6
Convolution reconstruction kernel	Bv36
Slice thickness (mm)	0.6
Bolus-tracking monitoring section	Auto trigger mode
Contrast agent	Ioversol (350 mg/ml)
Injection speed (ml/s)	3.5–4
Contrast agent volume (ml)	1.2–1.5/kg

### Digital design

**Figure 1:**
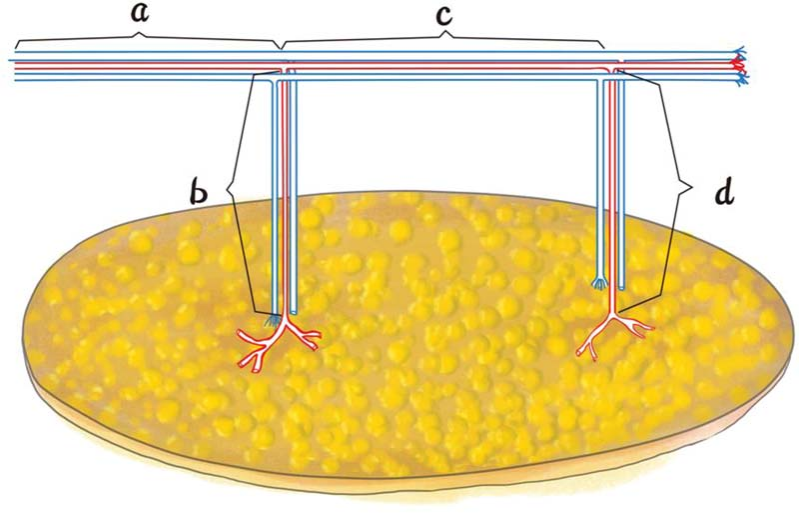
Schematic diagram of the vascular length of the skin flap: a is the length of the vascular pedicle; b + c + d is the length between selected perforators

(1) The original cross-sectional CTA image was observed on the PACS system, focusing on the vascular pedicle origin, number, position, type and length, and one thigh was selected as the donor site. The inclusion criteria were more than two perforators from the same source vessel with sufficient length between the selected perforators; if there were more than two perforators on both sides of the source artery, the vascular pedicle length and the perforator location and type were comprehensively considered. (2) The CTA data were imported into Mimics 20.0 software (Materialise, Belgian workstation) in DICOM format, and the source vessel, perforators, soft tissue and skeletal system of the selected thigh were reconstructed in 3D. (3) The anterosuperior iliac spine was used as a marker, and the vascular pedicle origin and length, perforator location and length between selected perforators were observed and measured with software. Two measurement methods were used for vessel length: the length of the vascular pedicle was defined as the distance from the emerging point to the origin of the source artery, and the length between selected perforators was defined as the length of the blood vessel between the ends of the first and second perforators (Figure 1). A free ALT lobulated perforator flap was designed in Mimics 20.0 based on factors such as wound size and shape and vascular anatomy so that the flap could completely cover the wound after repair, and the virtual operation was repeated to optimize the surgical plan.

### Flap harvesting

According to the preoperative digital design, the skin flap shape and vessels were drawn on the donor site, and the perforator locations and incision positions were marked. After the patient was placed in the supine position, the skin, subcutaneous tissue and deep fascia were cut along the inner edge of the flap, and the incision was extended to an appropriate length. In the fascia lata, 2–3 perforators that entered the deep fascia were located preoperatively, and these vessels were carefully protected. Then, the space between the rectus femoris and the lateral femoral muscle was found in the incision, the descending branch of the lateral circumflex femoral artery (d-LCFA) was separated and exposed to protect the nerve branches of the lateral femoral muscle, and dissection was continued to completely free the perforators. Then, the outer edge of the flap was cut to the deep fascia lata, and a small amount of skin at the distant proximal ends of the flap was kept connected to the distal and proximal ends of the thigh to prevent the vascular pedicle of the flap from overstretching. The skin flap was cut from the middle until it was completely removed. After the donor site completely stopped bleeding, the fascia lata was repaired and the wound was directly sutured.

According to the preoperative design, the flap was cut between the perforators and was converted into a lobulated flap. In the first stage, the edge of the flap was thinned and the lobulated flap was reconstructed in a shape similar to the wound surface in the recipient area. The flap was transplanted to the recipient area and sutured to the edge of the wound with multiple sutures, and the blood vessels in the recipient area were adjusted and anastomosed under the microscope. All of the included patients underwent anastomosis with the named artery and with two veins.

### Statistical analysis

Statistical analyses were performed using the SPSS software package (version 25.0; SPSS, Chicago, IL, USA). Kolmogorov–Smirnov analysis was used to assess the normality of the data distributions. All continuous variables are expressed as the mean ± standard deviation, and preoperative and intraoperative data were compared by paired t tests*.* A *p* value < 0.05 indicates statistical significance.

## Results

Digital reconstruction was successfully performed in all patients before surgery. The perforating vascular arteries in this group all originated from the d-LCFA. A total of 67 perforators were selected, including 17 septocutaneous perforators and 50 myocutaneous perforators. The length between the selected perforators was 8.2–11.2 cm (mean, 9.48 ± 0.83 cm), and the length of the vascular pedicle was 9–16.8 cm (mean, 12.16 ± 2.22 cm) before surgery. The 3D model was used to accurately guide the design and harvest of the flap during the operation. The flap harvesting time varied from 52–165 min (mean, 111.36 ± 34.31 min). The flap size ranged from 21 × 6.5 cm to 28 × 9 cm, and it was confirmed that the perforator number and type were the same as those determined before the surgery. During the surgery, the length between the selected perforators ranged from 7.9–11.5 cm (mean, 9.40 ± 0.89), and the length of the vascular pedicle was 9–16.8 cm (mean, 12.16 ± 2.22 cm). No significant differences (*p >* 0.05) were detected in the length of the vascular pedicle (*p* = 0.940, *t* = −0.075) or the length between selected perforators (*p* = 0.734, *t* = 0.341) before and during surgery. Except for 4 patients who survived after emergency re-exploration due to vascular crisis after surgery, all the flaps survived, and each donor site was directly closed. Nine patients underwent two rounds of thinning, 7 patients underwent one round of thinning and 12 patients did not undergo thinning (Table 3). The flap follow-up time ranged from 13–27 months (mean, 19.75 months). The skin flaps showed good color and texture. In patients with corresponding affected areas, shoes could be worn normally on the foot and ankle, and flexion and extension of the hand were normal, while flexion and extension of the elbow were slightly limited. All the donor sites exhibited linear scarring.

**Table 3:** Operative outcomes

	**Number**
Flap survival	28
Flap complications	
Arterial	1
Venous congestion	3
Re-exploration	4
Hematoma	0
Donor thigh	
Ipsilateral	13
Contralateral	15
Type of perforator	
Myocutaneous	21
Septocutaneous	7
Recipient artery	
Dorsalis pedis artery	10
Posterior tibial artery	4
Anterior tibial artery	3
Radial artery	11

### Case report

**Figure 2:**
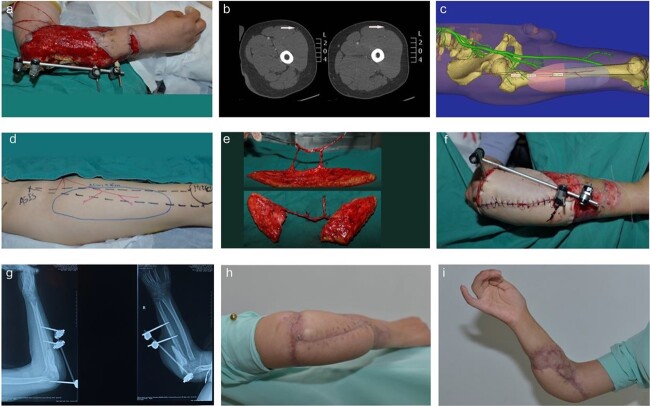
Repair of a large soft tissue defect in the right forearm with an ALT lobulated perforator flap. (**a**) Soft tissue defect and bone exposure in the right elbow. (**b**) Preoperative CTA examination revealed that there were two perforators at the donor site (white arrows). (**c**) Preoperative digital reconstruction and simulated flap harvesting. (**d**) According to the digital design, the skin flap was drawn on the donor site, and the perforator vessel and incision positions were marked on the surface. (**e**) Intraoperative flap harvest. (**f**) Wound repair. (**g**) Postoperative X-ray; vancomycin-loaded bone cement was used to fill the bone defect. (**h**, **i**) Postoperative view of the flap at the 19-month follow-up.* ALT* anterolateral thigh, *CTA* computed tomography angiography

**Case 1.** A 43-year-old male patient suffered from an open fracture of the right forearm caused by a car accident. In the first stage, emergency debridement, external fracture fixation and VSD placement were performed. Four weeks later, the soft tissue defects were wide and irregular, and the ulna showed a partial defect. To repair wide and irregular soft issue defects, we ultimately selected the ALT lobulated perforator flap. Before the operation, CTA and digital technology were used to reconstruct a 3D image of the d-LCFA. The results showed that the right d-LCFA had two good perforating branches, which agreed with the flap design principle. According to the shape and size of the patient's wound, a lobulated flap was designed with digital technology to simulate the position of the perforating branches of the d-LCFA, and the locations were marked on the donor site. The flap size was 25 × 8 cm, the flap was successfully harvested, and the wound was repaired during the operation. During the operation, the artery of the flap was anastomosed with the radial artery, vancomycin-loaded bone cement was used to fill the bone defect and the donor site was directly sutured. After intensive dressing changes and anti-infection treatment, the local distal infection improved. Satisfactory flap survival was observed at the 19-month follow-up assessment, with 120° of elbow flexion and 5° of elbow extension, indicating slightly limited elbow function (Figure 2).

**Figure 3:**
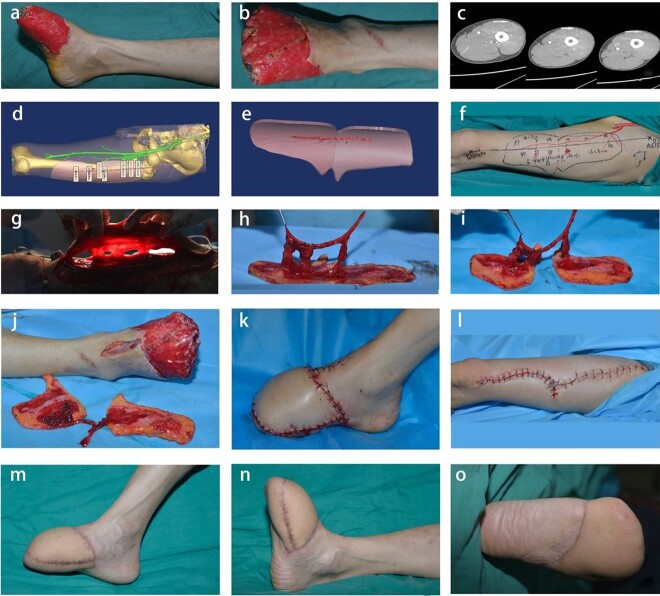
Repair of a large irregular soft tissue defect in the middle and distal right foot via the ALT lobulated perforator flap. (**a**, **b**) Appearance of soft tissue defects in the foot before the operation. (**c**) Preoperative CTA revealed that there were three perforators at the donor site (white arrows). (**d**, **e**) Preoperative digital reconstruction and simulated flap harvest. (**f**) According to the digital design, the skin flap was drawn on the donor site, and the perforator vessel and incision positions were marked on the surface. (**g**) Three perforators located before the operation were found by intraoperative exploration. (**h**, **i**) Intraoperative flap harvest. (**j**) The pedicle of the flap was anastomosed with the dorsalis pedis artery. (**k**, **l**) Primary repair of the wound surface and direct suturing of the donor site during the operation. (**m**–**o**) At follow-up, the foot and skin flap showed a good appearance. *ALT* anterolateral thigh, *CTA* computed tomography angiography

**Case 2.** A 36-year-old male patient was admitted to the hospital with avulsion of the middle and distal right foot caused by a car accident. Emergency debridement and VSD were performed after admission. Two weeks later, soft tissue defects in the right instep and sole were found, and toes 1–5 were missing. The area of the defect was 18 × 12 cm. Before the operation, CTA and digital technology were used to reconstruct a 3D image of the d-LCFA to clarify the course, distribution, location, number and relationship with adjacent tissues of the perforators. According to the shape and size of the patient’s wound, a lobulated flap was designed with digital technology to simulate the position of the perforator vessels of the d-LCFA, and the locations were marked on the donor site. During the operation, the location and course of the perforators were consistent with the preoperative design. The flap size was 28 × 9 cm. The artery and vein of the flap were anastomosed with the dorsalis pedis artery and its accompanying vein, and the donor site was directly sutured. The skin flap was not bloated in appearance and had good color and texture. The flexion and extension functions of the ankle joint were normal, and the donor site showed healing with a linear scar at the 21-month follow-up assessment (Figure 3).

## Discussion

Complex tissue defects caused by high-energy trauma are increasingly common; these defects can be large and deep and are often accompanied by tendon, bone, blood vessel and nerve damage and exposure. Additionally, the wound surface is often irregular, with different degrees of dead space. Improper treatment can lead to delayed wound healing, chronic osteomyelitis, amputation and other complications, which greatly reduce the quality of life of patients. These defects represent a substantial economic burden for countries and for society. It is difficult to treat these complex soft tissue defects in the clinic and the ideal method is repair via a perforator flap. The traditional method of directly creating a skin flap according to the shape of the defect wound makes directly suturing the donor site difficult, leading to the need for skin grafting and sacrifice of a second donor site. Moreover, scar hyperplasia and itching at the donor site aggravate the patient’s pain and discomfort. To design a composite skin flap for wound repair, it is necessary to anastomose two groups of blood vessels and sacrifice a second donor site. At present, there is a consensus on the need to protect donor sites, and direct suturing of donor sites in stage I can significantly reduce the occurrence of donor site complications [21]. Therefore, the ideal method of choice for repairing this kind of wound is to transplant a lobulated flap with lobes supplied by the same blood vessel into the recipient area and ensure direct suturing of the donor area in the first stage [18]. In this study, CTA was used to map all tissues at the donor site, and these images were subjected to digital reconstruction with computer software. Simulated surgery was then carried out according to the vascular anatomy and wound. An ALT lobulated flap, with the d-LCFA as the source artery and multiple perforators as the pedicle, was cut and prepared so that the flap was divided into parts, thus allowing the repair of complex, irregular and wide wounds while sacrificing only one donor site to repair the wound; this approach reduced the procedures needed for wound repair and maximized outcomes.

The management of blood vessels is very important for the survival of skin flaps. In our experience, we have found that to avoid damaging the perforator vessels of the flap, before the operation, digital technology is very useful for accurately locating the perforators and designing the flap. During surgery, medial and lateral incisions must be made first, and the skin and subcutaneous tissue between the two perforators must be cut under direct vision. After vascular anastomosis, the position of the vascular pedicle of each lobulated flap should be reasonably adjusted to avoid compression and distortion of the vessel, and the temperature and humidity of the operation area should be maintained during the perioperative period. Complete hemostasis should be achieved during the operation to avoid thrombosis and embolization after electrocoagulation hemostasis and prevent hematomas from forming and compressing the vascular pedicle, which could affect the survival of the flap. Only fascia lata within a radius of 3 cm centered on the perforating blood vessels needed to be transferred with the flap. The remaining fascia lata were kept in their original position as much as possible and were completely sutured after muscle recovery to avoid muscle herniation. While injury to the muscular branch of the femoral nerve should be avoided during the operation, most patients report some degree of sensory loss in the lateral thigh [22]. If the perforators are interwoven with the muscular nerve branches, they should be cut and repaired at the entry point of the muscle. When combining lobulated flaps, care should be taken to avoid several situations that promote perforator embolism. (1) Prevent each part of the flap from rotating more than 180° around the perforator. The management of blood vessels, even perforators, is highly important for the survival of skin flaps. (2) Avoid spiral twisting of the perforator in the subcutaneous tunnel. (3) Prevent the pedicle of the perforator from crossing rigid structures such as tendons and bones at a certain angle. (4) Avoid blood vessel penetration through a severely infected area. If any of the above situations occur, slight swelling, hematocele or inflammatory stimulation of the skin flap after the operation may lead to occlusion of the small perforator lumen, which will eventually lead to vascular embolism.

The advantages of the ALT lobulated perforator flap include the following. (1) The two flap lobes are supplied by the same source vessel and the recipient area requires only anastomosis of a group of blood vessels. (2) By adjusting the two lobes within a certain range, larger and more complicated wounds can be repaired. (3) Flap that can be harvest in a long length rather than a wide one can ensure direct closure of the donor site and reduce subsequent complications at the donor site. (4) The fascia lata can be repaired, which reduces damage to the donor site, promotes the formation of a linear scar at the donor site and improves the aesthetics of the donor site. The disadvantages of this flap include the following. (1) The flap has certain requirements in terms of the blood vessels at the donor site. (2) The primary blood vessels must have more than two relatively independent perforators, and each perforator must be able to form a relatively independent lobe. (3) There must be a certain length between the perforators to allow the two flap lobes to move relatively freely, and the blood supply of the flap must not be obviously damaged during lobulation. Our experience suggests that the minimum length between two perforators must be greater than the width of the skin flap, which is highly important. However, the number of perforating branches of ALT flaps varies greatly, and 18.2% of perforating branches are single perforating branches [17], which cannot be cut into lobulated flaps. Therefore, the failure rate of ALT lobulated perforator flap harvesting, according to experience, is high. Therefore, it is very important to accurately determine the number and position of perforators for an ALT lobulated perforator flap before surgery. In our study, we used digital technology to avoid the risks associated with vascular variation and achieve accurate preoperative vascular navigation. Moreover, the 3D model established before the operation can also be used by young doctors to learn the anatomy of the ALT flap and practice the surgical skills of flap harvesting.

Digital technology has been widely used in various fields of orthopedics [23–25] as it shows good potential for application in preoperative design and navigation and for playing an auxiliary role in complex flap surgery [26]. The design and harvest of skin flaps require a clear understanding of the location and shape of the perforator; thus, accurate localization of the perforator is key for successful skin flap transplantation. To date, many new vascular localization techniques have been applied in the clinic, and CTA and color Doppler ultrasound (CDU) have been widely used [27–33]. CDU examination is noninvasive and does not require the use of radiation or contrast agents. CDU can display hemodynamic information in real time and help to determine the quality of perforators. Additionally, compared with CTA, CDU is less expensive. However, this approach also has shortcomings, such as inspection results being highly dependent on the skills and experience of the operator. Furthermore, the resolution of CDU may not be as clear as that of CTA, especially for deep blood vessels, and CDU does not display the integrity and continuity of blood vessels as well as CTA. Thus, in the case of some anatomical variations, the necessary parameters may be more difficult to determine. CTA offers high contrast for blood vessels and can accurately reveal vascular anatomy, such as the shape, position and diameter of the perforating branches and the length of the available vascular pedicle; moreover, this method is objective and can quickly provide stable and reproducible images with high repeatability. The 3D structure generated by CTA can fully represent perforators. In this study, based on the original images obtained by CTA, d-LCFA and their perforators were reconstructed visually, and the design and harvest of the lobulated flap were virtually simulated with the software. The results showed that this digital technology had obvious advantages in repairing complex soft tissue defects of the limbs. There were substantial differences in the branches of the LCFA between the two sides, and the images obtained clearly displayed the shape and variation of the d-LCFA and its perforators. CTA can help in selecting the thigh that is more suitable as the donor site for the flap, thereby avoiding failed flap resection due to vascular variation and reducing the difficulty of surgery. This digital technology can display the 3D anatomical relationships of blood vessels and skin flaps intuitively, dynamically, stereoscopically and from multiple angles and can be used to reconstruct the target area in a concrete and personalized way to achieve the best functional and aesthetic effects. In addition, the length of the vascular pedicle and the distance between perforating branches can be measured before the operation, and doctors can perform virtual surgery before real surgery. The procedures for skin flap incision and skin flap combination can be simulated, increasing the accuracy of the operation. Such an accurate preoperative design can significantly shorten the operation duration and reduce both the number of blind procedures performed during the operation and perioperative complications. By displaying 3D models or predicted images, doctors can also explain the surgical process, expected results and potential risks to patients more clearly before the operation. The application of digital technology in flap design is reasonable and effective and can allow direct suturing of the donor site, further reducing damage to the donor site.

This study is limited by the small number of patients, and additional studies of more patients at multiple centers are needed to objectively observe the curative effect of this technology. In addition, CTA is still a relatively expensive examination, increasing the cost to patients, and there are disadvantages, such as exposure to radiation and certain potential contrast agent allergies.

## Conclusion

In this study, digital technology was used to assist in the design and harvest of ALT lobulated perforator flaps to achieve accurate localization before surgery, optimize the surgical plan, reduce surgical difficulty and risk, and shorten the operation time, providing a basis for the clinical design of personalized perforator flaps and highlighting the importance of the clinical application and promotion of this surgical method.

## Abbreviations

ALT: Anterolateral thigh; CTA: Computed tomography angiography; d-LCFA: Descending branch of the lateral circumflex femoral artery; CDU: Color Doppler ultrasound; VSD: Vacuum sealing drainage.

## Data Availability

The database used during the current study is available from the corresponding author upon reasonable request.

## References

[1] Hallock GG . Evidence-based medicine: lower extremity acute trauma. Plast Reconstr Surg. 2013;132:1733–41. 24281598 10.1097/PRS.0b013e3182a80925

[2] Kim CY , Kim YH. Supermicrosurgical reconstruction of large defects on ischemic extremities using supercharging techniques on latissimus dorsi perforator flaps. Plast Reconstr Surg. 2012;130:135–44. 22418715 10.1097/PRS.0b013e318254b128

[3] Wong CH , Ong YS, Wei FC. The anterolateral thigh - Vastus lateralis conjoint flap for complex defects of the lower limb. J Plast Reconstr Aesthet Surg. 2012;65:235–9. 21937295 10.1016/j.bjps.2011.08.043

[4] Zeiderman MR , LLQ P. Contemporary approach to soft-tissue reconstruction of the lower extremity after trauma. Burns Trauma. 2021;9:tkab024. 34345630 10.1093/burnst/tkab024 PMC8324213

[5] Liu Y , Song DJ, Xie SL, Song T, Zhang WT, Tian XN, et al. Clinical effects of free thinned deep inferior epigastric artery perforator flap in repairing extensive soft tissue defects in extremities. Chin J Burns. 2020;36:590–3. 10.3760/cma.j.cn501120-20190415-00185 32842406

[6] Li H , Xiao SE, Deng CL, Wu BH, Wu XK, Zhang TH, et al. Clinical application of combination of different types of free perforator flaps in the repair of complex wounds in extremities. Chin J Burns Wounds. 2023;39:758–64. 10.3760/cma.j.cn501225-20220720-00300 PMC11630271 37805787

[7] Abbassi O , Freer F, Sing QQY, Hoshimatsu H, Karakawa R, Song D, et al. Multi-pedicled long fasciocutaneous free flaps in complex lower extremity reconstruction. J Plast Reconstr Aesthet Surg. 2022;75:893–939. 10.1016/j.bjps.2021.11.097 34896041

[8] Kang Y , Pan X, Wu Y, Ma Y, Liu J, Rui Y. Subacute reconstruction using flap transfer for complex defects of the upper extremity. J Orthop Surg Res. 2020;15:134. 32264917 10.1186/s13018-020-01647-0 PMC7140501

[9] Olivan MV , Busnardo FF, Faria JC, Coltro PS, Grillo VA, Gemperli R. Chimerical anterolateral thigh flap for plantar reconstruction. Microsurgery. 2015;35:546–52. 26367370 10.1002/micr.22492

[10] Miyamoto S , Fujiki M, Nakatani F, Sakisaka M, Sakuraba M. Free flow-through anterolateral thigh flap for complex knee defect including the popliteal artery. Microsurgery. 2015;35:485–8. 25914181 10.1002/micr.22421

[11] Song YG , Chen GZ, Song YL. The free thigh flap: a new free flap concept based on the septocutaneous artery. Br J Plast Surg. 1984;37:149–59. 6713155 10.1016/0007-1226(84)90002-x

[12] Chana JS , Wei FC. A review of the advantages of the anterolateral thigh flap in head and neck reconstruction. Br J Plast Surg. 2004;57:603–9. 15380693 10.1016/j.bjps.2004.05.032

[13] Yang L , Cai B, Xue JR, Jiang P, Guo XZ. Clinical effects of individualized free anterolateral thigh flap in repairing complex refractory wound. Chin J Burns. 2020;36:730–4. 10.3760/cma.j.cn501120-20190621-00281 32829614

[14] Zhang W , Xie WG, Yang F, Zhang WD, Chen L. Clinical application of lobulated transplantation of free anterolateral thigh perforator flap in the treatment of electric burns of limbs. Chin J Burns. 2019;35:790–7. 10.3760/cma.j.issn.1009-2587.2019.11.005 31775467

[15] Di Candia M , Lie K, Kumiponjera D, Simcock J, Cormack GC, Malata CM. Versatility of the anterolateral thigh free flap: the four seasons flap. Eplasty. 2012;12:e21. 22582118 PMC3343765

[16] Qing LM , Wu PF, Yu F, Zhou ZB, Tang JY. Use of dual-skin paddle anterolateral thigh perforator flaps in the reconstruction of complex defect of the foot and ankle. J Plast Reconstr Aesthet Surg. 2018;71:1231–8. 30001914 10.1016/j.bjps.2018.05.029

[17] Lee YC , Chen WC, Chou TM, Shieh SJ. Anatomical variability of the anterolateral thigh flap perforators: vascular anatomy and its clinical implications. Plast Reconstr Surg. 2015;135:1097–107. 25502859 10.1097/PRS.0000000000001103

[18] Marsh DJ , Chana JS. Reconstruction of very large defects: a novel application of the double skin paddle anterolateral thigh flap design provides for primary donor-site closure. J Plast Reconstr Aesthet Surg. 2010;63:120–5. 19019746 10.1016/j.bjps.2008.08.022

[19] Li J , Luo X, Liu A, Zou Y. Clinical application of digital technology in the reconstruction of soft tissue defects of the lower extremity with free superficial circumflex iliac artery flap. Front Surg. 2022;9:956800. 36117845 10.3389/fsurg.2022.956800 PMC9478366

[20] Ni Y , Zhang X, Meng Z, Li Z, Li S, Xu ZF, et al. Digital navigation and 3D model technology in mandibular reconstruction with fibular free flap: A comparative study. J Stomatol Oral Maxillofac Surg. 2021;122:e59–64. 33242657 10.1016/j.jormas.2020.11.002

[21] Microsurgery Society of Chinese Medical Association . Guidelines for Clinical Application of MBCMA anterolateral femoral flap (Draft 2016). Chin J Microsurg. 2016;39:313–7.

[22] Kimata Y , Uchiyama K, Ebihara S, Sakuraba M, Lida H, Nakatsuka T, et al. Anterolateral thigh flap donor- site complications and morbidity. Plast Reconstr Surg. 2000;106:584–9. 10987464 10.1097/00006534-200009030-00009

[23] Chen YX , Zhang K, Hao YN, Hu YC. Research status and application prospects of digital technology in orthopaedics. Orthop Surg. 2012;4:131–8. 22927146 10.1111/j.1757-7861.2012.00184.x PMC6583460

[24] Knapp PW , Keller RA, Mabee KA, Pillai R, Frisch NB. Quantifying Patient Engagement in Total Joint Arthroplasty Using Digital Application-Based Technology. J Arthroplast. 2021;36:3108–17. 10.1016/j.arth.2021.04.022 33965282

[25] Wu X , Wang G, Xia Q, Rong K, Gan M, Wen G, et al. Digital Technology Combined with 3D Printing to Evaluate the Matching Performance of AO Clavicular Hook Plates. Indian J Orthop. 2020;54:141–7. 10.1007/s43465-019-00034-0 PMC7096338 32257030

[26] Chen YW , Yen JH, Chen WH, Chen IC, Lai CS, Lu CT, et al. Preoperative computed tomography angiography for evaluation of feasibility of free flaps in difficull reconstruction of head and neck. Ann Plast Surg. 2016;76:S19–24. 26808762 10.1097/SAP.0000000000000690

[27] Cohen OD , Abdou SA, Nolan IT, Saadeh PB. Perforator Variability of the Anterolateral Thigh Flap Identified on Computed Tomographic Angiography: Anatomic and Clinical Implications. J Reconstr Microsurg. 2020;36:616–24. 32643763 10.1055/s-0040-1713668

[28] Zhang YH , Cui WJ, Song KX, Sun LG, Wang F, Liu XZ, et al. A prospective study of the perforator evaluation and eccentric design of anterolateral thigh flap based on superficial fascial perforators assisted by modified computed tomography angiography. Chin J Burns Wounds. 2023;39:141–9. 10.3760/cma.j.cn501225-20220902-00376 PMC11630432 36878523

[29] Zhao SM , Liu YM, Liu N, Zhang HL, Song ZF, Gao WH, et al. Clinical effects of retrograde anterolateral thigh perforator flaps assisted with computed tomography angiography in repairing skin and soft tissue defects around the knee or in proximal lower leg. Chin J Burns. 2021;37:356–62. 10.3760/cma.j.cn501120-20200905-00401 PMC11917228 33874708

[30] Ma WG , Wang CD, Wang A, Liu F. Effect of free medial plantar perforator flap in repairing deep burn wound on palm with the assistance of three dimensional computed tomography angiography. Chin J Burns. 2020;36:323–6. 10.3760/cma.j.cn501120-20190308-00091 32340425

[31] Debelmas A , Camuzard O, Aguilar P, Qassemyar Q. Reliability of CDU Imaging for the Assessment of Anterolateral Thigh Flap Perforators: A Prospective Study of 30 Perforators. Plast Reconstr Surg. 2018;141:762–6. 29481406 10.1097/PRS.0000000000004117

[32] Gong J , Jia YP, Luo WD, Li CJ. Preoperative high-frequency CDU assessment of the blood vessels of the fibular myocutaneous flap. J Plast Reconstr Aesthet Surg. 2022;75:3964–9. 36216703 10.1016/j.bjps.2022.08.045

[33] Moore R , Mullner D, Nichols G, Scomacao I, Herrera F. CDU versus Computed Tomography Angiography for Preoperative Anterolateral Thigh Flap Perforator Imaging: A Systematic Review and Meta-Analysis. J Reconstr Microsurg. 2022;38:563–70. 34959247 10.1055/s-0041-1740958

